# Stereotactic body radiotherapy for patients with non-small-cell lung cancer using RapidArc delivery and a steep dose gradient: prescription of 60% isodose line of maximum dose fitting to the planning target volume

**DOI:** 10.1093/jrr/rry112

**Published:** 2019-01-22

**Authors:** Yuichiro Tsurugai, Atsuya Takeda, Naoko Sanuki, Takahisa Eriguchi, Yousuke Aoki, Yohei Oku, Takeshi Akiba, Akitomo Sugawara, Etsuo Kunieda

**Affiliations:** 1 Radiation Oncology Center, Ofuna Chuo Hospital, 6-2-24 Ofuna, Kamakura, Kanagawa, Japan; 2 Department of Radiation Oncology, Tokai University School of Medicine, 143 Shimokasuya, Isehara, Kanagawa, Japan

**Keywords:** stereotactic body radiotherapy, early-stage non-small-cell lung cancer, local control, prescription isodose line, age-adjusted Charlson comorbidity index

## Abstract

We retrospectively investigated outcomes, including pulmonary toxicities, of stereotactic body radiation therapy using RapidArc and a risk-adapted 60% isodose plan for early-stage non-small-cell lung cancer patients. We evaluated patients staged as cT1a–2bN0M0 between 2011 and 2017 and treated with a total dose of 40–60 Gy in five fractions to the 60% isodose line of the maximum dose encompassing the planning target volume with curative intent. Comorbidities and age were rated using an age-adjusted Charlson comorbidity index (AACCI). Factors associated with overall survival (OS) were investigated. A total of 237 patients with 250 lesions were eligible. The median follow-up was 28.0 months. The local recurrence rate at 3 years was 0.8%; none of the patients developed isolated local recurrence. OS, deaths from lung cancer, and deaths from intercurrent disease at 3 years were 72.7%, 8.2% and 19.1%, respectively. On multivariate analysis for correlating factors with OS, AACCI and maximal standardized uptake value on [18F]-fluorodeoxyglucose positron emission tomography/computed tomography remained significant. Grade ≥3 toxicities were limited to radiation pneumonitis in six (2.4%) patients (Grade 3 in four patients and Grade 5 in two patients). Among those, three patients had idiopathic interstitial pneumonia. The total dose was unrelated to the incidence of Grade ≥3 radiation pneumonitis (*P* = 0.69). Using the 60% isodose prescription and RapidArc, maximal local control was achieved with acceptable toxicities. Although the OS may depend on patient background, dose escalation aiming at higher local control can be beneficial for medically inoperable patients.

## INTRODUCTION

Stereotactic body radiotherapy (SBRT) is a standard treatment for patients with medically inoperable early-stage non-small-cell lung cancer (NSCLC). Although many studies have reported its good local control, SBRT delivery methods, including immobilization, total dose, fractionation, and prescription, vary among institutions.

We have treated patients with NSCLC using SBRT risk-adapted at the 80% isodose line of the maximum dose to the planning target volume (PTV) since 1998. In 2010, we reassessed the optimal prescription isodose line encompassing the PTV. We found that the 60% isodose plan led to improved dosimetry to normal lung tissue, with higher comparative mean PTV and internal target volume (ITV) doses achieved, along with good conformity index values. Based on the results, we performed a feasibility study for patients with peripheral lung tumors using a total dose of 60 Gy in five fractions at the 60% isodose line, with a maximum dose to the PTV of 100 Gy, and confirmed that the 60% isodose plan is feasible in the acute and subacute phase. In addition, we started using volumetric-modulated arc therapy (VMAT) with RapidArc (Varian Medical Systems, Palo Alto CA, USA) SBRT. Following these changes, we have treated patients with lung tumors with SBRT and a risk-adapted prescription using a 60% isodose plan. The aim of this study was to retrospectively investigate outcomes and pulmonary toxicities of SBRT using VMAT and a risk-adapted 60% isodose plan.

## MATERIALS AND METHODS

### Patients

Among consecutive lung cancer patients treated with SBRT in our hospital between May 2011 and September 2017, we retrospectively identified patients staged as cT1a–2bN0M0 using the 8th edition of the AJCC and Union for International Cancer Control (UICC) staging system guidelines, who were treated with a total dose of 40–60 Gy in five fractions to the 60% isodose line of the maximum dose encompassing the PTV with curative intent. Most patients underwent [18F]-fluorodeoxyglucose positron emission tomography/computed tomography (18F-FDG-PET/CT) for staging unless they had lung tumors characterized by a pure ground-glass opacity (GGO) appearance (the tumors have an extremely low level of SUVmax and a very low incidence of nodal and distant metastasis [1]). For patients without pathologic diagnosis due to technical or clinical difficulties with biopsy, our multidisciplinary lung cancer board review deemed the pulmonary tumor to be lung cancer based on clinical information such as an increase in the maximum standardized uptake value (SUVmax) on 18F-FDG-PET/CT, serial tumor enlargement and radiological findings on CT, and elevated tumor marker levels. Patients who had <6 months follow-up without death were excluded, as were patients with small-cell lung cancer.

Patients’ comorbidity was rated using the Charlson comorbidity index (CCI), a weighted index of comorbidity for 19 clinical conditions. Although several studies reported an association between CCI and mortality, the index does not include the effect of increasing age. A combined age–comorbidity scale, age-adjusted CCI (AACCI)], was subsequently validated, which adjusts for age by adding one point to the index score for each decade of life over 50. In the present study, AACCI was used to compare overall survival and cause of death between two groups divided at the sample median value.

All patients’ data were routinely recorded and retrospectively reviewed for this study. This retrospective study was approved by The Ofuna Chuo Hospital review board (No. 2018-003). At treatment, written informed consent was obtained from all patients for permission of treatment data utilization in future retrospective studies.

### Treatment

We have previously reported the details of our SBRT technique. After immobilization using a vacuum pillow and abdominal compression corset, long-scan-time CT for treatment planning was acquired to directly visualize the ITV. The PTV was determined by adding 6–8 mm margins to the ITV. For SBRT delivery, non-coplanar VMAT plans were created using the Eclipse treatment planning system (version 10.0; Varian Medical Systems) with the Acuros XB algorithm for heterogeneity correction. All plans were generated and delivered based on 6-MV photon beams from a Varian CLINAC iX linear accelerator equipped with 5 mm multileaf collimators (MLCs). Patient-specific partial arcs of >600°(cumulative for all beams) were designed for a highly conformal prescription dose distribution. In most cases, two coplanar arcs and two non-coplanar arcs with couch angles of 45°/315° were used. We used three different risk-adapted regimens based on tumor location: 60 Gy for peripherally located lesions non-adjacent to the chest wall or mediastinum; 50 Gy for peripherally located lesions adjacent to the chest wall or mediastinum and for centrally located lesions not including mainstem bronchus and/or main pulmonary artery within the PTV; and 40 Gy for centrally located lesions including the organs at risk (OARs) within the PTV. The doses were prescribed to the 60% isodose line of the maximum dose covering the PTV in five fractions. The corresponding maximum doses were 100 Gy, 83 Gy and 67 Gy in total prescribed doses of 60 Gy, 50 Gy and 40 Gy, respectively. Our institutional policy requires that 98% of the PTV receives 100% of the prescribed dose while adhering to the dose constraints of OARs using VMAT. We kept maximum doses to the volume of esophagus, spinal cord, and trachea with a 3-mm margin within 25 Gy. The ratio of lung volume receiving 20 Gy to total lung was ≤15%, and the volume of the brachial plexus receiving >50 Gy was ≤1 ml. No specific dose limits for heart and aorta were applied. A cone-beam CT scan was acquired just prior to each treatment delivery.

### Follow-up

Our follow-up procedures were previously described in detail. In brief, all patients were followed up monthly with interviews, laboratory data review, and chest X-ray examinations or high-resolution CT scans during the first 6 months. CT scans were scheduled at 1 and 3 months after SBRT and at 3-month intervals during the first 2 years thereafter. Subsequently, follow-up interviews and CT scans were obtained at 4- to 6-month intervals. In addition, 18F-FDG-PET/CT and brain magnetic resonance imaging were performed 1 year after SBRT or when recurrence was highly suspected.

### Statistical analysis

Local recurrence was defined as recurrence within or adjacent to the PTV and was diagnosed by pathological confirmation or serial enlargement that could not clearly be attributable to lung fibrosis on serial CT scans. We also used 18F-FDG PET/CT findings when recurrence was highly suspected. Other recurrences were defined as regional, with failure in both hila, mediastinal, or the supraclavicular fossa; or distant, with failure at other sites.

Local, regional and distant recurrences were calculated with a cumulative incidence function, accounting for death as a competing risk. Overall survival (OS) was estimated by the Kaplan–Meier method, and differences between groups were assessed using the log-rank test. Deaths from lung cancer and deaths from intercurrent disease were calculated with a cumulative incidence function, accounting for death from the other as a competing risk, and were compared using Gray’s test. Adverse events were graded using the Common Terminology Criteria for Adverse Events, version 4.

All statistical analyses were two-sided; *P* values <0.05 were considered statistically significant. Analyses were performed using EZR (Saitama Medical Center, Jichi Medical University), which is a graphical user interface for R (The R Foundation for Statistical Computing, version 3.4.1). More precisely, it is a modified version of R commander (version 2.4–0) that includes statistical functions that are frequently used in biostatistics.

## RESULTS

### Eligible patients

Between May 2011 and September 2017, 240 patients receiving SBRT for lung cancer with a total dose of 40–60 Gy in five fractions at the 60% isodose line of the PTV with curative intent were staged as cT1a–2bN0M0. Among these patients, three were excluded for small-cell carcinoma. A total of 237 patients with 250 lesions were eligible for this study. Thirteen lesions, which were treated with a second SBRT, were diagnosed as second primary lung cancers. Table 1 shows patients, tumors and treatment characteristics. The median follow-up time was 28.0 months (range, 0.8–76.9 months) for all patients and 31.0 months (range, 6.0–76.9 months) for surviving patients. Only one patient was lost to follow-up at 40.6 months after SBRT.

**Table 1.** Patient, tumor and treatment characteristics

Patient characteristics		*n* = 237
Age, median (range)		79 (54, 93)
Sex	Male/female	162/73
CCI, median (range)		2 (0, 10)
AACCI, median (range)		5 (2, 13)
Operability	Yes/no	66/171
GOLD	Non-COPD/I/II/III/IV	127/42/33/27
Tumor and treatment characteristics		*n* = 250
T_8th	Tis/1mi/1a/1b/1c/2a/2b	9/1/15/76/59/84/6
Histology	Ad/Sq/NSCLC/unproven	69/20/17/144
SUV max, median (range)		3.10 (0.00, 30.10)
Location	Peripheral/central	204/46
Total dose	40/50/60	27/157/66
ITV (cm^3^), median (range)		7.70 (0.20, 52.10)
PTV (cm^3^), median (range)		33.19 (6.10, 124.60)
Mean ITVdose (Gy), median (range)		77.68 (58.65, 96.12)
Min ITVdose (Gy), median (range)		66.70 (43.37, 84.43)
Mean PTVdose (Gy), median (range)		65.20 (35.97, 82.90)
Min PTVdose (Gy), median (range)		43.79 (16.82, 72.04)
MLD (Gy), median (range)		3.71 (1.06, 7.86)
V20 (Gy), median (range)		4.59 (0.77, 13.05)

CCI = Charlson comorbidity index; AACCI = age-adjusted Charlson comorbidity index; GOLD = global initiative for chronic obstructive lung disease; COPD = chronic obstructive pulmonary disease; Ad = adenocarcinoma; Sq = squamous cell carcinoma; NSCLC = non-small-cell carcinoma; unproven; pathologically unproven; SUV = standardized uptake value; ITV = internal target volume; PTV = planning target volume; MLD = mean lung dose; V_20_ = lung volume irradiated with ≥20 Gy.

### Oncological outcomes

**Fig. 1.**
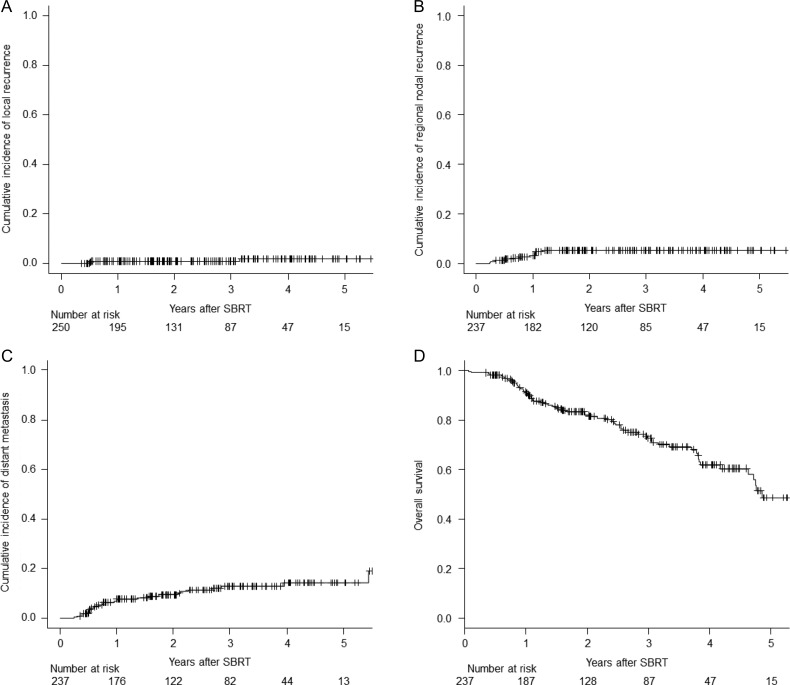
(A) Cumulative incidence of local recurrence for all patients. (B) Cumulative incidence of regional nodal recurrence for all patients. (C) Cumulative incidence of distant metastasis for all patients. (D) Overall survival curve for all patients.

At the time of analysis, 66 patients were dead and only one patient was lost to follow-up. None of the patients developed isolated local recurrence after SBRT. Three patients developed local recurrence, which was simultaneous with, or followed, regional recurrence or distant recurrence. Those patients died due to multiple metastases within a year of initial recurrence. The local recurrence rates at 3 years from the start of SBRT was 0.8% [95% confidence interval (CI): 0.2–2.8] for all patients. (Fig. 1A, cumulative incidence curve of local recurrence). The regional nodal recurrence and distant metastasis rates at 3 years were 5.1% (95% CI: 2.7–8.7) and 12.9% (95% CI: 8.5–18.2), respectively (Fig. 1B–C). The OS at 3 years was 72.7% (95% CI: 65.3–78.9) (Fig. 1D).

Table 2 shows the results of univariate and multivariate analyses of factors potentially related to OS. Univariate analyses indicated that AACCI, global initiative for chronic obstructive lung disease (GOLD) classification, T stage, SUVmax, age, sex, and operability were significantly associated with OS. On multivariate analysis, AACCI, GOLD classification and SUVmax remained significant factors. AACCI was the strongest predictor related to OS (*P* < 0.01).

**Table 2.** Univariate and multivariate analyses affecting OS

Variates	Univariate analysis			Multivariate analysis		
	HR	95% CI	*P* value	HR	95% CI	*P* value
Age	1.05	1.01–1.09	0.02			
Sex			0.04			0.99
Female	1			1		
Male	1.86	1.03–3.35		0.99	0.51–1.92	
AACCI			<0.01			<0.01
≤5	1			1		
≥6	3.07	1.79–5.24		2.93	1.63–5.29	
Operability			0.01			0.53
Yes	1			1		
No	2.44	1.28–4.66		1.25	0.62–2.51	
GOLD			<0.01			0.09
Non-COPD	1			1		
GOLD I–II	1.31	0.72–2.38		0.99	0.52–1.89	
GOLD III–IV	2.83	1.60–5.02		1.95	1.01–3.73	
T stage			<0.01			0.22
Tis-1c	1			1		
T2a-b	2.12	1.30–3.44		1.39	0.82–2.38	
Histology						
Proven	1		0.69			
Unproven	0.91	0.56–1.47				
SUV max			<0.01			0.02
≤2.55	1			1		
>2.55	2.28	1.32–3.95		2.00	1.11–3.62	
Location						
Central	1		0.43			
Peripheral	0.80	0.47–1.38				
Total dose (Gy)						
40	1		0.39			
50	0.66	0.35–1.27				
60	0.64	0.32–1.28				

AACCI = age-adjusted Charlson comorbidity index; GOLD = global initiative for chronic obstructive lung disease; COPD = chronic obstructive pulmonary disease; SUV = standardized uptake value.

**Fig. 2.**
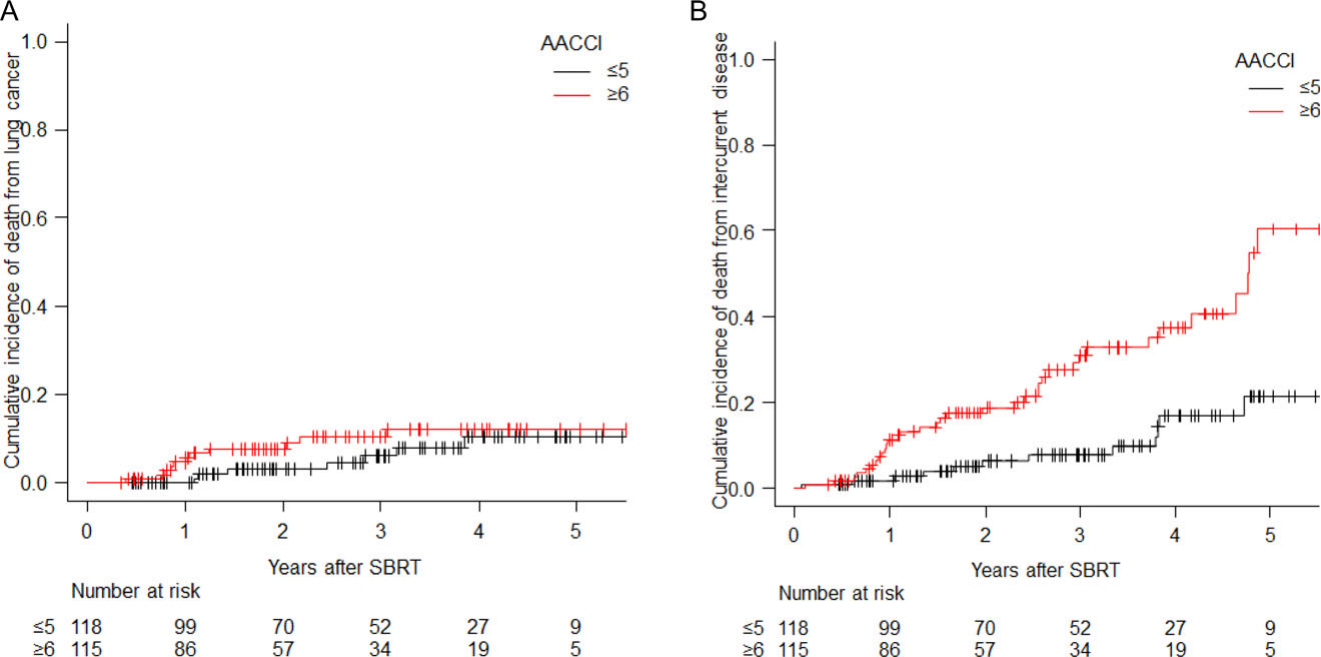
Cumulative incidence of death from lung cancer (A) and death from intercurrent disease (B) divided by AACCI. Abbreviation: AACCI: Age-adjusted Charlson comorbidity index.

For patients with AACCI of ≤5 vs those with AACCI of ≥6, no significant difference in cumulative incidence of death from lung cancer was seen, with rates at 3 years of 6.1% vs 10.3%, *P* = 0.30 (Fig. 2A). Death from intercurrent disease was statistically more frequent in patients with AACCI of ≥6 (7.7% vs 30.9%, *P* < 0.01) (Fig. 2B).

### Treatment-related toxicities

Toxicities ≥ Grade 2 were observed in 28 patients, including radiation pneumonitis in 24 (9.6%), chest wall pain in 3 (1.2%) and acute esophagitis in 1 (0.4%), respectively. Grade ≥ 3 toxicities were limited to radiation pneumonitis in six (2.4%) patients (Grade 3 in four patients and Grade 5 in two patients), including three patients having idiopathic interstitial pneumonia as comorbidities. One of two patients who had Grade 5 radiation pneumonitis had had underlying idiopathic interstitial pneumonitis. Grade ≥ 3 radiation pneumonitis occurred in 0.9% of patients without a history of idiopathic interstitial pneumonias. Total dose was not related to the incidence of Grade ≥ 3 radiation pneumonitis (*P* = 0.69).

## DISCUSSION

SBRT has seen increased sophistication of treatment apparatus and treatment planning equipment, and it is still progressing. The stereotactic techniques and high dose per fraction were initially used in gamma knife radiosurgery for brain tumors, and those concepts were applied to the lung in the 1990s. Many SBRT studies have reported local control rates of ≥90% for patients with primary early-stage lung cancer, comparable with surgery [2]. Many techniques in SBRT are undergoing improvement, including four-dimensional computed tomography simulation, dose prescription methods, dose calculation algorithms, and image guidance technology. By using those technologies, we are aiming at higher local control and safer treatment.

In SBRT, total dose and fractionation are among the most fundamental factors that influence local control. However, even if total dose and fractionation are kept the same, actual dose to the tumor depends on dose prescription points. In the isodose prescription plan, the dose is prescribed to the isodose line covering the PTV. Prescription to a lower percentage isodose line leads to a more inhomogeneous dose to the PTV and a higher dose to the center of the PTV where the tumor exists with high probability. In addition, the dose just outside of the PTV decreases steeply. ESTRO ACROP consensus guidelines reported that a majority of institutions prescribe a dose to the PTV periphery (PTV encompassing isodose or volumetric prescription D_99–95%_), and maximum PTV doses range between ≤125% and ≤150% of the prescription dose. In our previous study of isodose level comparative planning, we found the 60% isodose prescription was optimal among alternative 20–90% isodose plans. We subsequently conducted a Phase 1 study of SBRT using a 60% isodose, and revealed the method was feasible. Following those studies, we have applied the 60% isodose prescription to clinical SBRT since 2011, achieving extremely high local control and safety using the isodose prescription.

Onishi *et al.* reported good local control with a prescribed biologically effective dose (BED) ≥100 Gy in SBRT for patients with early-stage lung cancer. However, it should be noted that the analysis was conducted without considering differences in prescription points and methods among institutions. In fact, some institutions prescribed a maximum dose with homogenous dose distribution in the PTV, while others prescribed an 80% isodose covering the PTV periphery, suggesting almost minimum dose to the PTV. Considering the uncertainties in BED comparison, some studies using BED have recently been conducted to investigate which BED index with which definition correlates with better local control: ITV dose coverage (BED10 > 150 Gy) , maximum dose and mean PTV (BED10 > 125 Gy). In this study, the dose was prescribed at the 60% isodose line of the maximum dose covering the PTV (our institutional policy requires that 98% of the PTV receive 100% of the prescribed dose). Among the risk-adapted three dose groups (levels) with 60% isodose plan, the lowest prescribed dose was 40 Gy in five fractions (BED 10 = 72 Gy). According to the ‘BED ≥100 Gy’ principle, it seemed to be insufficient for SBRT with curative intent. However, the maximum dose to the PTV, the mean ITV dose and the mean PTV dose were ~67 Gy (BED 10 = 157 Gy), 62 Gy (BED 10 = 139 Gy) and 53 Gy (BED 10 = 109 Gy), respectively. Those values indicate that a high dose to the actual tumor may have resulted in no local recurrence, even in the 25 patients receiving 40 Gy/five fractions.

VMAT is a novel planning and delivery method in the category of intensity-modulated arc therapies aiming to deliver highly modulated plans with variable MLC configurations, dose rate, and gantry speed during rotation. The features also contribute to high local control in SBRT for lung cancer. It can generate steep dose gradients and fit irregularly shaped PTVs. Some studies have reported that VMAT planning showed more favorable target dose conformity than multiple static fields planning in SBRT for early-stage lung cancer. Doses to the OARs can also be diminished. Rauschenbach *et al.* showed that VMAT enabled dose–volumetric factors to the OARs to be lower compared with three-dimensional conformal radiotherapy (3DCRT); including mean lung dose, lung volumes irradiated with ≥20 Gy, and the maximum dose to the ipsilateral brachial plexus, proximal bronchial tree, esophagus, heart, and spinal cord. Although there is concern that VMAT may increase the incidence of radiation pneumonitis by increasing the lung volume receiving a low dose, such as lung volumes irradiated with ≥5 Gy, Palma *et al.* reported that no difference was seen in the rate of Grade 2/3 radiation pneumonitis between 3DCRT and VMAT. Also in this study, the frequencies of radiation pneumonitis were not high: Grade ≥2 was 9.6% and Grade ≥3 was 2.4%. In addition, among the patients not having idiopathic interstitial pneumonias, Grade ≥3 radiation pneumonitis occurred only in 0.9%. The frequencies were even lower than those reported by previous studies, despite the fact that the lung within the PTV was irradiated with extremely high doses and the lung volume receiving at least a low dose was extensive. In our institution, we have used clarithromycin to prevent and treat radiation pneumonitis in some high-risk patients, exploiting its immunomodulatory and anti-inflammatory effects, and found that it may mitigate radiation pneumonitis following SBRT. That may partially explain the low rates of severe radiation pneumonitis.

It is true, of course, that OS depends on patient background, including age and coexisting diseases, as well as on the biological activity of the lung cancer as suggested by SUVmax, which we already reported was a significant prognostic factor. We therefore adopted AACCI as an objective tool for predicting OS for patients treated with SBRT. As a result, AACCI was the strongest OS predictor in multivariate analysis; for patients with AACCI ≥ 6 vs those with AACCI ≤ 5, a 3-year cumulative incidence of death from intercurrent disease was four times higher (30.7% vs 7.7%, *P* < 0.01). The indication for sublobar resections as a treatment option for early-stage NSCLC with patients having mild or medically well-controlled comorbidities is increasing. However, these comorbidities can cause postoperative complications and adversely affect prognosis. SBRT provides good oncological outcomes with less toxicity for frailer patients, as shown in this study. With the increased number of aging lung cancer patients, multidisciplinary discussions based on evaluation of the coexisting comorbidities, the morbidity risk following treatment, and performance status have been important factors in the selection of optimum treatment.

As the standard treatment for inoperable NSCLC Stage I patients changed from conventional radiotherapy to SBRT, local control rates dramatically improved and survival consequently improved. Again, to investigate dose–response curves in SBRT, early data demonstrated that BED10 > 100 Gy resulted in significantly better survival. More recent studies have suggested that even further dose escalation of BED10 beyond 125 or 150 Gy correlates with higher local control. However, other researchers wondered if small improvements in local tumor control from 90% to 95% led to improved survival, especially in medically inoperable patients at high risk from competing causes of death. In this study, 3-year local control improved from 93% to 99% compared with our previous results using an 80% isodose prescription, whereas, the OS was not improved significantly (73% in the current study vs 78% in our previous study). Nonetheless, similar OS between the two studies could potentially be due to distant metastases being the predominant recurrence pattern, and to recently expanded indications (e.g. older and frail patients, or more advanced cases). However, with expanding treatment indications becoming available to operable patients with ≥T2 tumors, dose escalation aiming at higher local control using a 60% isodose prescription and the VMAT method would be more beneficial.

This study has the usual limitations of all single-institutional retrospective series, including the potential for selection bias, although we had collected data prospectively, and followed up frequently over the long term with only one patient lost to follow-up. The small population of central lung cancer cases (*n* = 46), may have weak power to derive a conclusion regarding appropriate dose and fractionation for the central tumor. Although AACCI is a strong indicator for OS, it has also limitations. For example, scores of comorbidities (e.g. myocardial infarction, diabetes, chronic lung disease) listed as scores on CCI are too simple to reflect the severity or prognosis of each disease.

## CONCLUSION

Using a 60% isodose prescription and VMAT, extremely high local control and safety were achieved. Although the OS may depend on patient background, dose escalation aiming at higher local control can be beneficial for medically operable patients. Further prospective studies are warranted to investigate the safety and effect of dose escalation in SBRT for early-stage NSCLC.
